# Inflammatory profiles across the spectrum of disease reveal a distinct role for GM-CSF in severe COVID-19

**DOI:** 10.1126/sciimmunol.abg9873

**Published:** 2021-03-10

**Authors:** Ryan S Thwaites, Ashley Sanchez Sevilla Uruchurtu, Matthew K Siggins, Felicity Liew, Clark D Russell, Shona C Moore, Cameron Fairfield, Edwin Carter, Simon Abrams, Charlotte-Eve Short, Thilipan Thaventhiran, Emma Bergstrom, Zoe Gardener, Stephanie Ascough, Christopher Chiu, Annemarie B Docherty, David Hunt, Yanick J Crow, Tom Solomon, Graham P Taylor, Lance Turtle, Ewen M Harrison, Jake Dunning, Malcolm G Semple, J Kenneth Baillie, Peter JM Openshaw, on behalf of the ISARIC4C investigators

**Affiliations:** 1 National Heart and Lung Institute, Imperial College London, U.K.; 2 University of Edinburgh Centre for Inflammation Research, Edinburgh, U.K.; 3 Dept of Clinical Infection, Microbiology and Immunology, University of Liverpool, U.K.; 4 Centre for Medical Informatics, Usher Institute, University of Edinburgh, Edinburgh, U.K.; 5 Centre for Genomic and Experimental Medicine, MRC Institute of Genetics and Molecular Medicine, University of Edinburgh, Edinburgh, U.K.; 6 Department of Infectious Disease, Faculty of Medicine, Imperial College London, U.K.; 7 Intensive Care Unit, Royal Infirmary Edinburgh, Edinburgh, U.K.; 8 Centre for Clinical Brain Sciences, University of Edinburgh, Edinburgh, U.K.; 9 Tropical and infectious disease unit, Liverpool University Hospitals NHS Foundation Trust (member of Liverpool Health Partners), U.K.; 10 National Infection Service, Public Health England, London, UK; 11 NIHR Health Protection Research Unit in Emerging and Zoonotic Infections, Institute of Infection, Veterinary and Ecological Sciences, Faculty of Health and Life Sciences, University of Liverpool, Liverpool, U.K.; 12 Respiratory Medicine, Alder Hey Children’s Hospital, Liverpool, U.K.; 13 Roslin Institute, University of Edinburgh, Edinburgh, U.K.

## Abstract

While it is now widely accepted that host inflammatory responses contribute to lung injury, the pathways that drive severity and distinguish coronavirus disease 2019 (COVID-19) from other viral lung diseases remain poorly characterized. We analyzed plasma samples from 471 hospitalized patients recruited through the prospective multicenter ISARIC4C study and 39 outpatients with mild disease, enabling extensive characterization of responses across a full spectrum of COVID-19 severity. Progressive elevation of levels of numerous inflammatory cytokines and chemokines (including IL-6, CXCL10, and GM-CSF) were associated with severity and accompanied by elevated markers of endothelial injury and thrombosis. Principal component and network analyses demonstrated central roles for IL-6 and GM-CSF in COVID-19 pathogenesis. Comparing these profiles to archived samples from patients with fatal influenza, IL-6 was equally elevated in both conditions whereas GM-CSF was prominent only in COVID-19. These findings further identify the key inflammatory, thrombotic, and vascular factors that characterize and distinguish severe and fatal COVID-19.

## Teaser

GM-CSF is elevated early, scaled with severity, and is central to the inflammatory response in COVID-19, but not severe influenza.

## INTRODUCTION

Fatal COVID-19 is associated with acute respiratory distress syndrome (ARDS) and elevated markers of systemic inflammation including IL-6 and C-reactive protein (CRP), often accompanied by peripheral blood neutrophilia and lymphopenia (*1*). However, IL-6 concentrations are typically tenfold lower than those reported in ARDS and sepsis, and other mediators also have major roles in pathogenesis (*2*–*5*). The beneficial effect of corticosteroids (*6*, *7*) and IL-6 receptor antagonists (*8*) in severe COVID-19 indicates that immune inhibition can be beneficial at advanced stages of disease and that inflammation is a modifiable component of COVID-19 pathogenesis. With many biologic therapies to choose from, it is important to establish which additional pathways and mediators should now be prioritized in clinical trials. Identification of inflammatory mediator profiles reflective of processes that are associated with disease severity may define ‘treatable traits’ (*9*), allowing both stratification of patients likely to benefit from therapies such as dexamethasone and targeted biological anti-cytokine therapies, and design of novel therapeutics targeting causative pathways.

An influx of monocytes/macrophages into the pulmonary parenchyma and a myeloid pulmonary artery vasculitis have been reported in autopsy studies of COVID-19. In addition, critical illness in COVID-19 is associated with genotype-inferred CCR2 expression in the lung and there is strong evidence that myeloid cells contribute to immunopathology (*10*–*14*). In addition to macrovascular thrombosis (*15*), pulmonary microthrombosis is a frequent autopsy finding with additional evidence of endothelial injury and endotheliitis, implicating endothelial activation and coagulation in respiratory failure (*10*, *11*, *13*, *16*). The virus-induced inflammatory state has laboratory features that resemble secondary haemophagocytic lymphohistiocytosis (sHLH) (*17*–*19*) but the exact pattern and severity of inflammatory responses has only been partially characterized. Other host factors also influence COVID-19 severity, with polymorphisms in interferon pathway genes *IFNAR2* and *OAS1/2/3* associating with variations in disease severity (*14*).

Early clinical studies of COVID-19 showed elevated neutrophil counts and lymphopenia in peripheral blood (*1*, *20*), especially in late-stage disease, though neutrophilia is commonly seen in other severe respiratory viral (*21*) and bacterial (*22*) infections. Elevated levels of D-dimer, a product of fibrin-degradation associated with thrombosis and inflammation, have also been observed in COVID-19 (*20*), consistent with systemic inflammation and the high frequency of intravascular thrombotic complications (*13*, *23*). Thromboses and pulmonary microthrombi are common in fatal COVID-19 and are associated with endothelial responses distinct from those that occur during fatal influenza A virus infection (*10*–*12*, *24*). However, the thrombotic aspects of life-threatening COVID-19 have hitherto been described in relatively small groups of cases, from single-center studies, or with a narrow range of disease severities.

The exceptional scale and range of ISARIC4C study allows us to examine a wide range of responses that reflect the spectrum of COVID-19 disease severity. We also analyzed and compared selected samples from patients with severe influenza from the 2009-10 A/H1N1 pandemic, enabling comparative analysis and the identification of unique aspects of COVID-19 pathogenesis that may be amenable to therapeutic manipulation.

## RESULTS

### Routine clinical data did not completely distinguish COVID-19 severities

We obtained clinical data and plasma samples from 471 patients hospitalized with COVID-19 within the ISARIC4C study (*25*, *26*) using a publicly available protocol as a pre-positioned pandemic preparedness study (*26*, *27*). Patients were stratified into five clinical groups based on their peak illness severity according to the World Health Organization COVID-19 ordinal scale (*28*) (table S1): (1) no oxygen requirement (Severity 3, n=132); (2) patients requiring oxygen by face mask or nasal prongs (Severity 4, n=106); (3) patients requiring high-flow nasal cannulae oxygen or non-invasive ventilation (Severity 5 n=79); (4) patients requiring invasive mechanical ventilation (Severity 6/7, n=85); and (5) fatal COVID-19 (Severity 8, n=69).

The median duration of symptoms prior to recruitment and sample collection was 9 days, but differences were evident between severity groups: Severity 3, 7 days; Severity 4, 10 days; Severity 5, 11 days; Severity 6/7, 12 days; and Severity 8, 11 days. These differences were significant between groups 3 and 5 (*P*=0.0003) and groups 3 and 6/7 (*P*=0.0030) (fig. S1A), possibly reflecting the recruitment of patients with mild symptoms that were hospitalized for monitoring or isolation early in the pandemic. The median duration between admission and study completion (discharge or death) was 9 days (IQR 6-18; range 1-89).

Some differences in routinely performed clinical hematology and biochemistry measures were evident between clinical groups at enrolment: Lymphopenia was present in groups 6/7 and 8, relative to 3, and neutrophilia in 6/7 and 8 relative to 3 and 4 (fig. S1B and S1C, respectively). No differences between groups were observed in ferritin levels (elevated in most patients), while LDH was elevated in groups 5, 6/7, and 8 relative to 3 (fig. S1D and S1E, respectively). Procalcitonin levels were elevated in group 8 relative to groups 3, 4, and 5 (all *P*<0.05, fig. S1F). Partial HScores (*29*) were calculated (fever, cytopenia, ferritin, triglycerides, and AST) but the only significant difference between groups was between severities 6/7 and 4, indicating that sHLH-like disease is unlikely to be the predominant pathophysiological mechanism in life-threatening COVID-19 (fig. S1G). The ISARIC4C mortality scores (*30*) for these patients demonstrated a stepwise increase with each increment in severity and, as expected, were highest in patients that would progress to fatal disease (group 8) relative to all other groups (fig. S1H).

### A broad inflammatory response scaled with COVID-19 severity

**Fig. 1:**
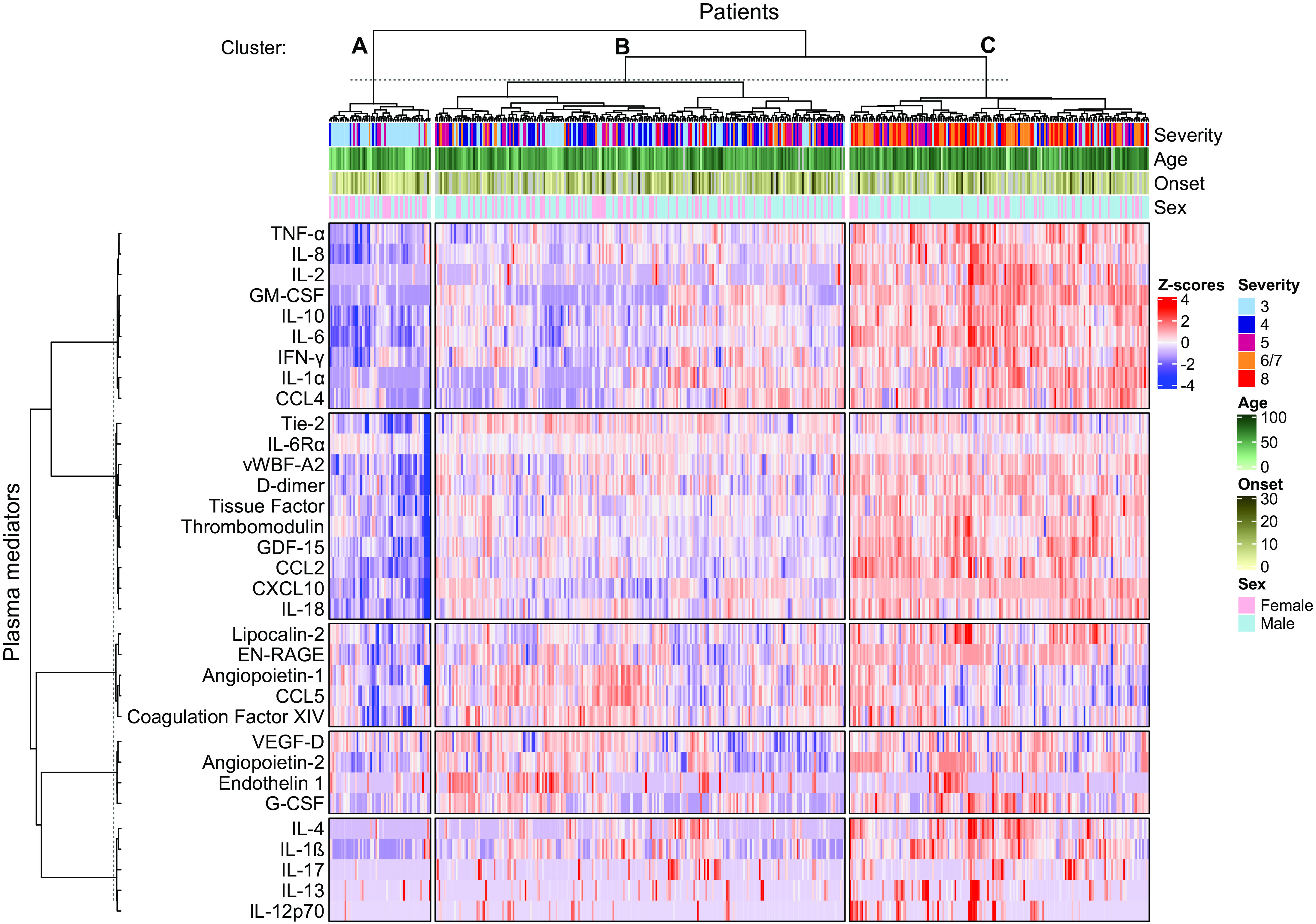
Plasma mediators at the time of study enrollment demonstrated a broad exaggerated immune response in patients hospitalized with COVID-19. Clustered heatmap of 33 immune mediators in plasma samples collected from patients hospitalized with COVID-19 at the time of study enrolment. Missing mediator data were imputed and values were scaled within each mediator. Rows and columns were split by K-means clustering. Each patients’ column is additionally annotated with data on disease outcome (“Severity”) as one of the following outcome groups: not requiring oxygen support (‘3′, n=132), requiring oxygen via face mask or nasal prongs (‘4’, n=106), requiring non-invasive ventilation or high-flow nasal canulae oxygen (‘5′, n=79), requiring invasive mechanical ventilation (‘6/7’, n=85) or fatal disease (‘8’, n=69). Columns are additionally annotated with patient age, sex and duration of illness at the time of sample collection (“Onset”).

To determine the relationship between levels of plasma markers and disease severity, we used immunoassays to measure 33 mediators chosen to represent putative mechanistic pathways of disease (*20*, *23*). Data were hierarchically clustered and annotated with the severity, age, disease duration at the time of sampling (“Onset”), and sex of each patient. This analysis identified 3 clusters of patients with distinct patterns of mediators, which fell into 5 hierarchical clusters (Fig. 1).

The three clusters of patients had different clinical characteristics (table S2). Cluster A (n=59) had milder illness (with no deaths and 76.3% of patients from severity group 3), were more commonly female (55.9%), had a lower median age (54.7 years) and lower rates of diabetes mellitus (15.3%) than other clusters. Cluster A was also associated with lower levels of most mediators (Fig. 1). Patients in cluster C (n=174) had a substantially greater severity of illness (32.2% fatal; 33.9% mechanical ventilation), higher routine inflammatory markers (neutrophils and CRP) and temperature, and lower lymphocyte counts (median 0.8x10^9^/L). These patients were also older (median 64.1 years), predominantly male (74.1%) and were more likely to have diabetes mellitus (32.6%). This cluster was associated with the highest levels of many mediators including TNF-ɑ and IL-6. Patients in cluster B (n=238) had an intermediate clinical phenotype relative to clusters A and C and a more mixed profile of immune mediators.

Principal component analysis (PCA) was used to determine the main drivers in the variance between individuals, again using only plasma mediator data but annotating the PCA plot with patient disease severity. This analysis demonstrated considerable overlap between disease severity groups but some distinction between milder and more severe COVID-19, largely determined by PC1 (fig. S2A). The top 5 drivers of this variance (determined by PC1 loading values) were IL-6 (0.256), CXCL10 (0.254), GDF-15 (0.246), GM-CSF (0.241), and CCL2 (0.239) (fig. S2B).

Therefore, at the time of enrolment different COVID-19 outcome groups were identifiable based on distinct patterns of inflammatory mediators; IL-6, CXCL10, and GM-CSF were key determinants of cluster assignment.

### Myeloid and vascular inflammatory markers distinguished hospital and community managed COVID-19

**Fig. 2:**
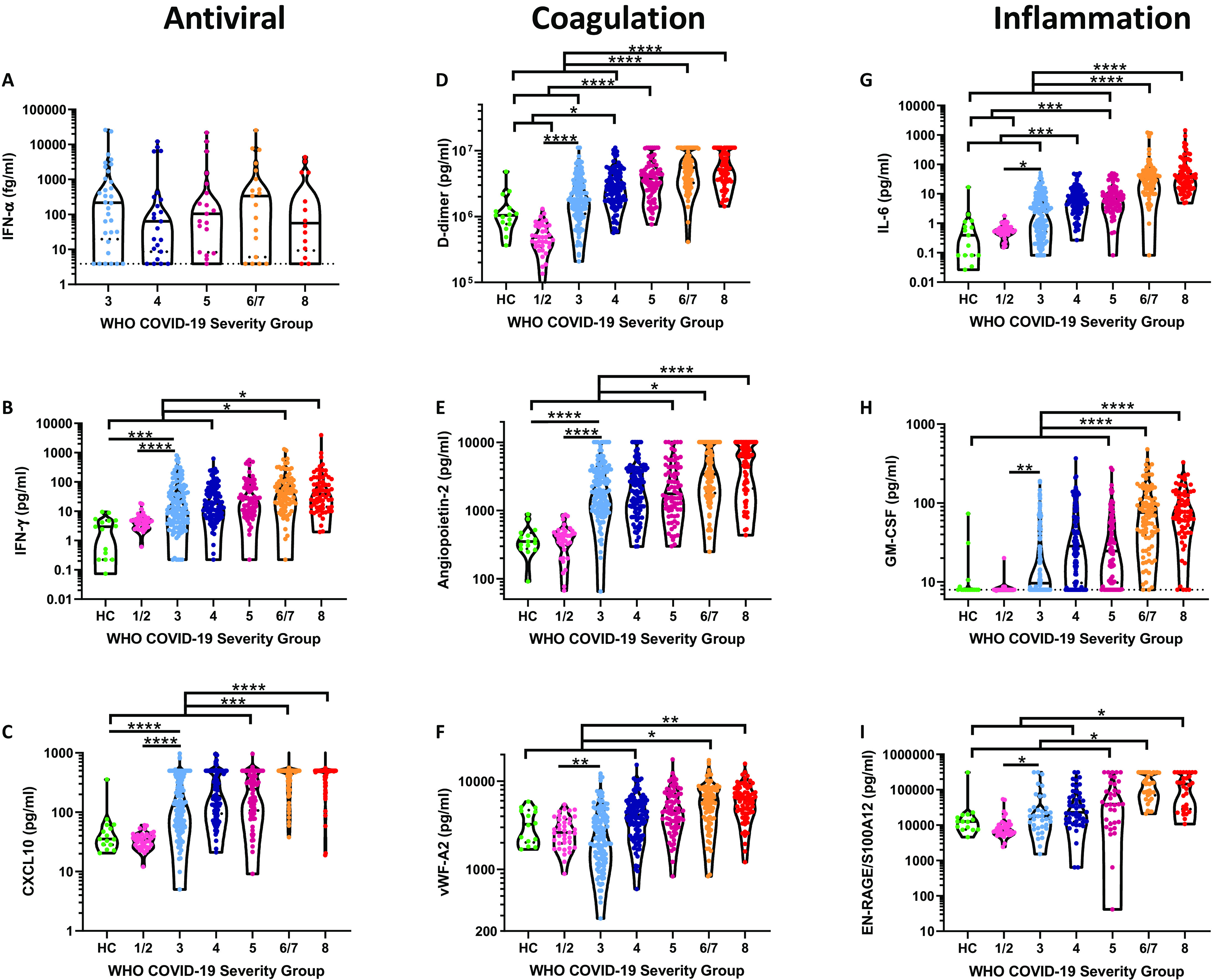
Antiviral, coagulation, and inflammation associated mediators distinguished severity groups early in disease. Plasma samples from the time of study enrolment were analyzed for levels of the antiviral cytokines **(A)** IFN-α, **(B)** IFN-γ, and **(C)** the interferon-induced chemokine CXCL10 in healthy control (HC, n=15), patients with COVID-19 not requiring hospitalization (‘1/2’, n=39), and hospitalized patients with COVID-19 that would: not require oxygen support (‘3′, IFN-α n=32, other mediators n=132), require an oxygen face mask (‘4’, IFN-α n=23, other mediators n=106), require non-invasive ventilation or high-flow nasal cannulae (‘5′, IFN-α n=19, other mediators n=79), require invasive mechanical ventilation (‘6/7’, IFN-α n=19, other mediators n=85) or progress to fatal disease (‘8’, IFN-α n=14, other mediators n=69). Mediators associated with coagulation and endothelial injury were also quantified in these plasma samples; **(D)** D-dimer, **(E)** Angiopoietin-2, and **(F)** von-Willebrand factor A2 (vWF-A2). Similarly, mediators associated with inflammation were quantified: **(G)** IL-6; **(H)** GM-CSF; and **(I)** EN-RAGE/S100A12. Violin plots display medians (solid lines) and interquartile ranges (dashed lines). Data were analyzed for statistical significance using Kruskal-Wallis tests with Dunn’s tests for multiple comparisons between all groups. *P<0.05, **P<0.01, ***P<0.001, ****P<0.0001.

To further explore the relationship between mediator levels and severity we analyzed plasma from 15 healthy controls (‘HC’; 7 males, median age 55, range 45-71) and 39 individuals recruited 7 days after a positive SARS-CoV-2 PCR test who did not require hospitalization (15 males, median age 43, range 27-62, termed group ‘1/2’ per the WHO scale (*31*)) and related these to hospitalized patients. Numerous differences were evident between hospitalized COVID-19 patients, outpatients, and healthy controls, along with many differences across the clinical groups in hospitalized patients (Fig. 2 and fig. S3). In contrast to other reports (*32*), we found no association between IFN-α levels and disease severity (Fig. 2A), when analyzed in a random subset of patient samples. IFN-γ was elevated in hospitalized COVID-19 patients relative to HC and group 1/2 (Fig. 2B) and was elevated in the most severe outcome groups, relative to lower severity grades. The interferon-induced chemokine CXCL10 was also substantially elevated in all hospitalized COVID-19 cases relative to the control groups, with the most pronounced increases in groups 6/7 and 8 (Fig. 2C). These results contrasted with decreased interferon stimulated gene expression in peripheral blood samples from patients with severe COVID-19 (*32*). These differences led us to speculate that the abundance of IFN-γ and CXCL10 resulted from release at the site of disease rather than from circulating cells, though anti-IFN autoantibodies (*33*) and polymorphisms in IFN signaling (*14*) may have influenced this pathway.

The fibrin degradation product D-dimer is elevated in severe COVID-19 (*20*), implicating thrombosis in disease severity, consistent with autopsy findings (*10*, *11*, *24*). In agreement with these reports, D-dimer was elevated in all hospitalized groups, with stepwise increases between severity groups (Fig. 2D). Given reports of the association between COVID-19 mortality and pulmonary vasculitis (*10*), we hypothesized that endothelial injury may be a feature of COVID-19, potentially triggering coagulation and the thrombotic complications common in severe disease (*23*, *34*). Indeed, levels of angiopoietin-2, a marker of endothelial injury, were elevated in all hospitalized patients relative to both control groups (Fig. 2E), with levels 5.6-fold higher in the mildest hospitalized patients (severity 3, median=1983pg/ml) than HCs (median=352pg/ml). Angiopoietin-2 levels were also significantly elevated in groups 6/7 and 8 relative to all other hospitalized COVID-19 outcome groups (Fig. 2E). As both angiopoietin-2 and vWF-A2 can enter the blood plasma through exocytosis of endothelial cell Weibel-Palade bodies (*35*), we also quantified vWF-A2 and Endothelin-1, which were similarly elevated in patients with severe COVID-19 (Fig. 2F and fig. S3, respectively). Elevations in these prothrombotic mediators were not counteracted by the inhibitors angiopoietin-1 or soluble Tie2, which were equivalent between groups (fig. S3). These results suggest that endothelial injury and coagulation are common features of patients hospitalized with COVID-19 and that these are most pronounced in severe and fatal COVID-19.

In line with other reports (*1*, *3*), we found that IL-6 was significantly elevated in most hospitalized groups relative to controls (Fig. 2G), with a stepwise increase in levels with escalating severity. IL-6 levels in groups 6/7 and 8 were significantly elevated above all other groups (all *P*<0.0001, Fig. 2G). GM-CSF was similarly elevated in all hospitalized groups relative to controls and was most pronounced in the groups 6/7 and 8 (Fig. 2H). Numerous other inflammatory cytokines and chemokines showed similar results including TNF-α, IL-2, GDF-15, G-CSF, and VEGF-D (fig. S3). EN-RAGE/S100A12 has previously been characterized as a biomarker of inflammation in ARDS (*36*) and indeed was elevated in groups 6/7 and 8 relative to most others (Fig. 2I). The neutrophil chemokine IL-8 (CXCL8) was similarly elevated in severe disease, as was the neutrophil gelatinase associated lipocalin (LCN-2/NGAL) (fig. S3), in line with the reported association between blood neutrophilia and severity (*20*) also seen in this cohort (fig. S1C).

In agreement with the PCA, CCL2 and GDF-15 were increased in groups 6/7 and 8 while other immunological mediators (IL-6Rα, IL-13, IL-17) were not significantly different between groups (fig. S3), indicating that only limited aspects of the immune repertoire were active in COVID-19. Interestingly, IL-4 levels were lower in the moderate/non-severe disease outcome groups (3, 4, and 5) relative to both control groups and severe disease groups (fig. S3), indicating that suppression of normal type-II cytokines levels may be associated with milder COVID-19, and that this is lost in severe disease. These data partially recapitulate the association of type 2 mediators with COVID-19 severity (*37*), though levels are typically low and require careful interpretation relative to control samples. Similarly, IL-12p70, commonly released by antigen presenting cells (APCs) (*38*), was decreased in all hospitalized cases relative to the HCs and group 1/2 (fig. S3). These results demonstrated that numerous mediators distinguished COVID-19 disease severity groups, yet many mediators remained equivalent between COVID-19 patients and controls, indicating that a broad, yet specific, inflammatory response contributes to immunopathogenesis.

### The inflammatory response in COVID-19 was coordinated around IL-6 and GM-CSF

**Fig. 3:**
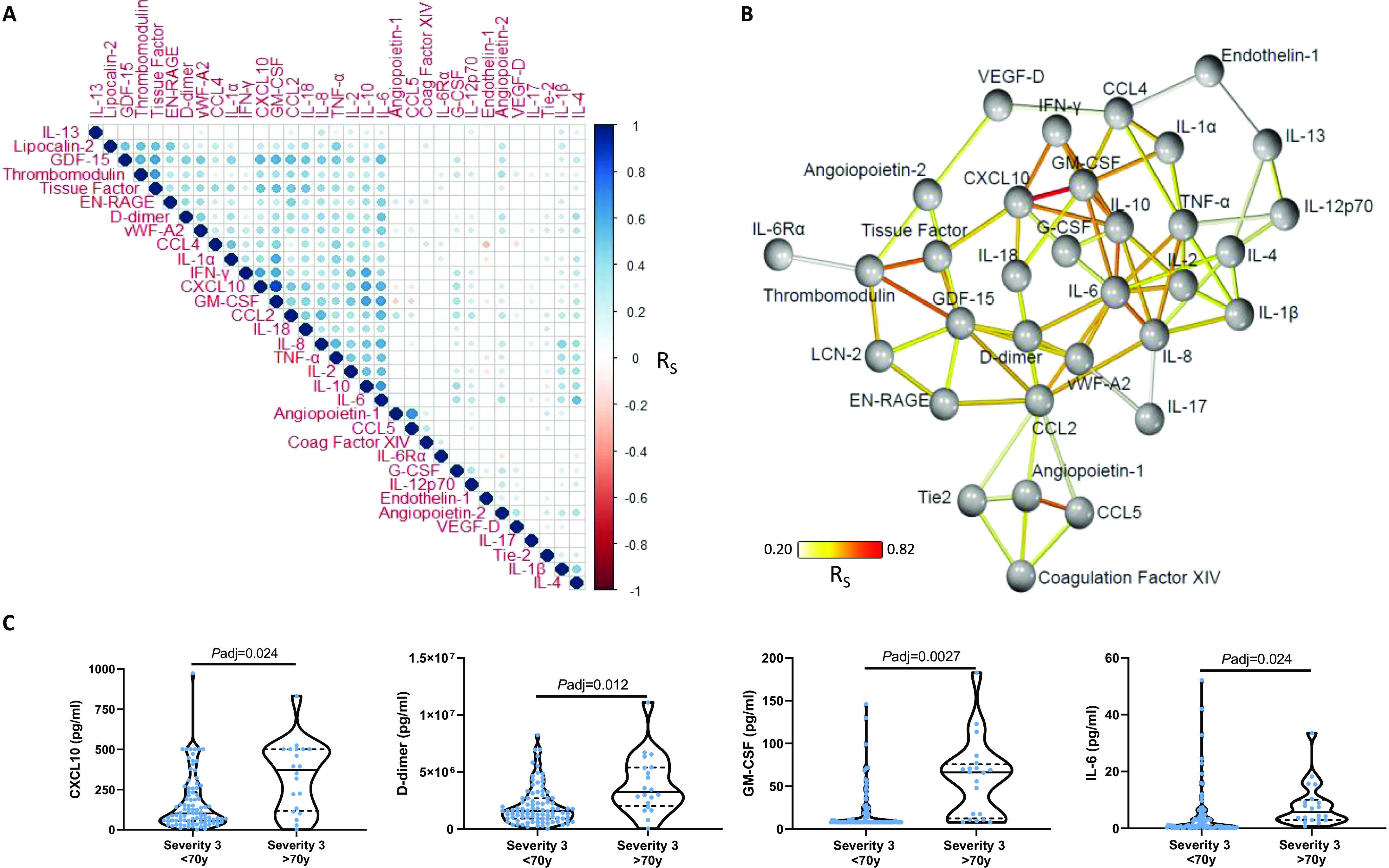
**Plasma mediators in COVID-19 were coordinated around IL-6 and GM-CSF and influenced by age. (A)** Correlogram of the association between plasma mediator levels at the time of enrolment in all patients hospitalized with COVID-19 (n=465). **(B)** Network analysis showing the mediator-to-mediator correlation profile. Nodes represent mediators and the coloring of edges between nodes represents the Spearman correlation coefficient (R_S_) connecting them. **(C)** Inflammatory mediator levels within an outcome group, stratified as those ≥ or < than 70 years of age. Data in panel a were analyzed using Spearman’s rank correlations with correction for multiple testing; significant correlations are denoted by a circle, the color of which denotes the Spearman’s R value. Data in panel C were analyzed using Mann-Whitney U tests with *P*-value adjustment for false discovery rate. Violin plots display medians (solid lines) and interquartile ranges (dashed lines).

PCA demonstrated that some specific inflammatory mediators (e.g., IL-6, CXCL10, GDF-15, GM-CSF, CCL2) were the strongest determinants of the variance apparent between COVID-19 patients (fig. S2). To determine the strength of the relationships between these plasma mediators, we performed a hierarchical correlation matrix analysis of mediator data. This identified a strongly correlated group of inflammatory mediators (including GM-CSF, CXCL10, D-dimer, vWF-A2, and IL-6; Fig. 3A), increases of which were associated with the most severe COVID-19 outcome groups (Fig. 2 and fig. S3). The overlap between these mediators and those identified by PCA indicated a coordinated myeloid and vascular inflammatory response associated with disease severity.

Using network analyses structured on the correlation values between mediator pairs, several inflammatory mediators (including GM-CSF, IL-2, and IL-6) grouped together and closely correlated with a wider group of inflammatory mediators (Fig. 3B and interactive 3D visualization: https://isaric4c.net/networks/). The antiviral mediators IFN-γ and CXCL10 were associated with this inflammatory mediator group, and there were close associations between many inflammatory mediators and markers of vascular and thrombotic responses (particularly D-dimer and vWF-A2). Network analyses therefore indicated a close association between the inflammatory and thrombotic elements of the immune response during COVID-19.

### Age, but not sex, influenced plasma mediator levels

Given the strong association between age and COVID-19 severity (*26*), and reports of increased inflammatory responses in males with COVID-19 (*39*) we investigated the influence of age and sex on plasma mediators levels. As the major effect in our cluster analysis was severity (Fig. 1), we further stratified each of these severity groups by age (≥ or < 70 years of age) and sex.

After adjustment for multiple testing, no mediator was found to be statistically different between males and females within each severity group (fig. S4). By contrast, several differences were evident between those aged ≥70 and <70 years within individual severity groups, with elevated levels of CXCL10, D-dimer, GM-CSF, IL-1ɑ, IL-6, IL-8, LCN-2, and TNF-ɑ in those aged ≥70 years (Fig. 3C and fig. S4); by contrast, IFN-γ in severity group 4 was the only mediator significantly elevated in younger patients (fig. S4).

### Early inflammatory mediator elevations in severe COVID-19

**Fig. 4:**
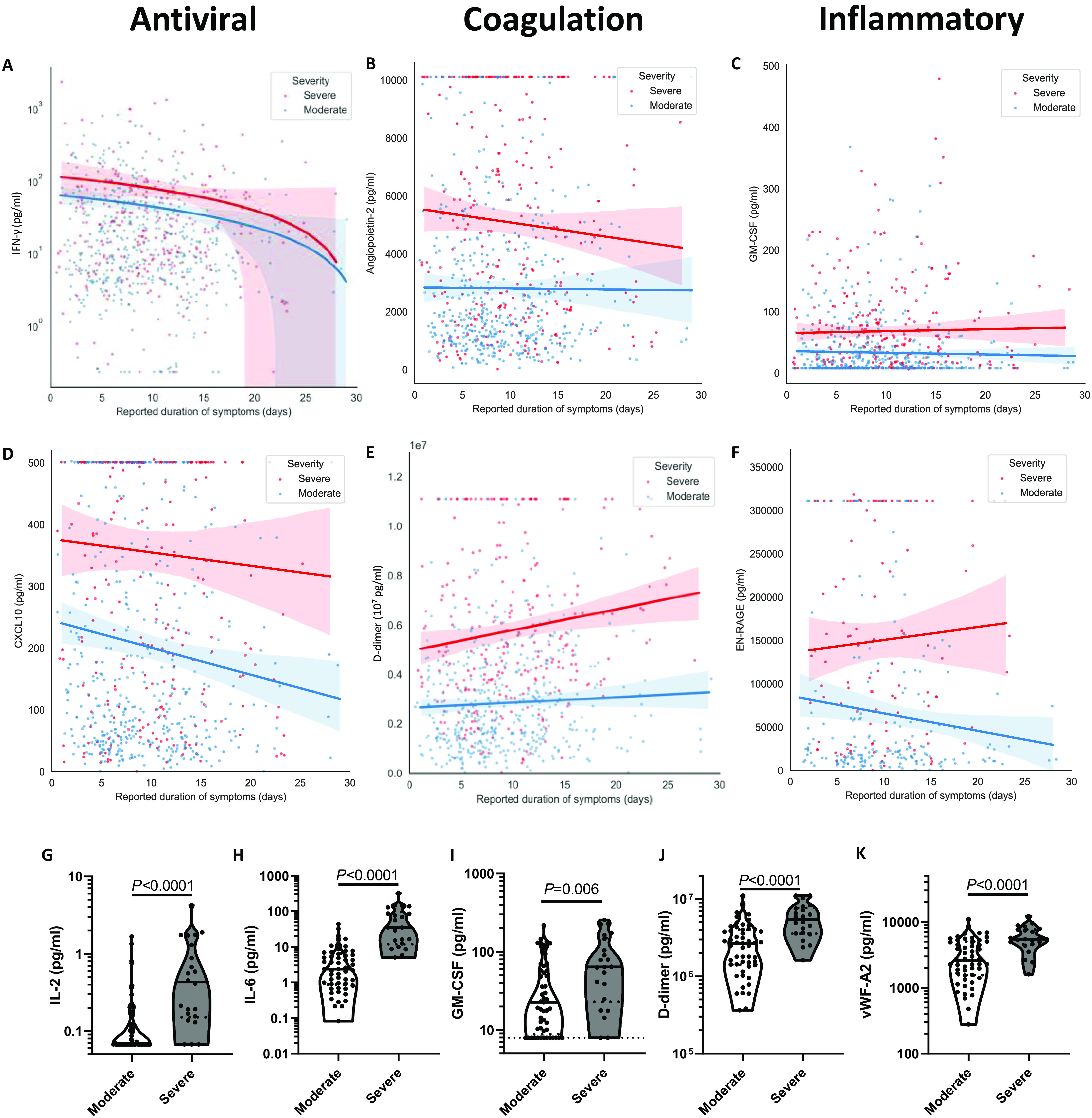
Longitudinal analysis of plasma mediator levels demonstrated a progressive immune response and an exaggerated inflammatory signature in fatal COVID-19. Plasma levels of **(A)** IFN-γ, **(B)** CXCL10, **(C)** Angiopoietin-2, **(D)** D-dimer, **(E)** GM-CSF, and **(F)** EN-RAGE/S100A12 over the course of disease in patients with fatal COVID-19. Plasma mediator levels of **(G)** IL-2, **(H)** IL-6, **(I)** GM-CSF, **(J)** D-dimer, and **(K)** von-Willebrand factor A2 (vWF-A2) within the first 4 days of symptom onset in patients in severity groups 6/7 or 8 (“Severe”, n=22) and groups 3, 4, or 5 (“Moderate”, n=54). Linear regressions with 95% confidence intervals are shown in panels A-F. Data in panels G-K were analyzed for statistical significance using Mann-Whitney U tests, where solid lines denote the median values and dashed lines denote the interquartile ranges.

We next sought to identify changes in the levels of plasma mediators over the course of disease by relating mediator levels to the patient reported duration of symptoms at the time of sampling. For this exploratory analysis patients were grouped into ‘Moderate’ (severities 3, 4, and 5; n=317) and ‘Severe’ (severities 6/7 and 8; n=154) outcome categories. Many mediators apparently remained largely stable over time, including IFN-γ, angiopoietin-2, and GM-CSF (Fig. 4A-C, respectively). By contrast, there was a slight decrease over time of CXCL10 levels (Fig. 4D), an increase over time in D-dimer (Fig. 4E), and an increase over time in S100A12/EN-RAGE in the Severe, but not the Moderate, category (Fig. 4F). Most other tested mediators were largely stable over time (fig. S5).

Given the stability over time of those mediators most closely associated with disease severity (including IL-6 and GM-CSF), we hypothesized that differences in plasma mediator levels between patients with severe and moderate COVID-19 would be apparent early in the course of disease. Indeed, within the first 4 days of symptoms several mediators were significantly elevated in the severe group, relative to those with moderate disease, including IL-2, IL-6, and GM-CSF (*P*<0.0001, *P*<0.0001, and *P*<0.006, Fig. 4G-I, respectively), indicating a pronounced inflammatory response early in severe disease. Similarly, many markers of coagulation and endothelial injury were elevated in severe disease, relative to moderate, including D-dimer and vWF-A2 (*P*<0.0001, Fig. 4J and 4K, respectively), in addition to angiopoietin-2 and IL-1α (which can be activated by thrombin (*40*)) (fig. S6). By comparison the lung damage-associated marker EN-RAGE (*36*) was not significantly different between the severe and moderate groups in the first 4 days of symptoms (*P*=0.098, fig. S6), though time course data indicated this mediator may be elevated in the later stages of severe disease (Fig. 4F).

### GM-CSF and IL-1α distinguished fatal COVID-19 from fatal influenza

**Fig. 5:**
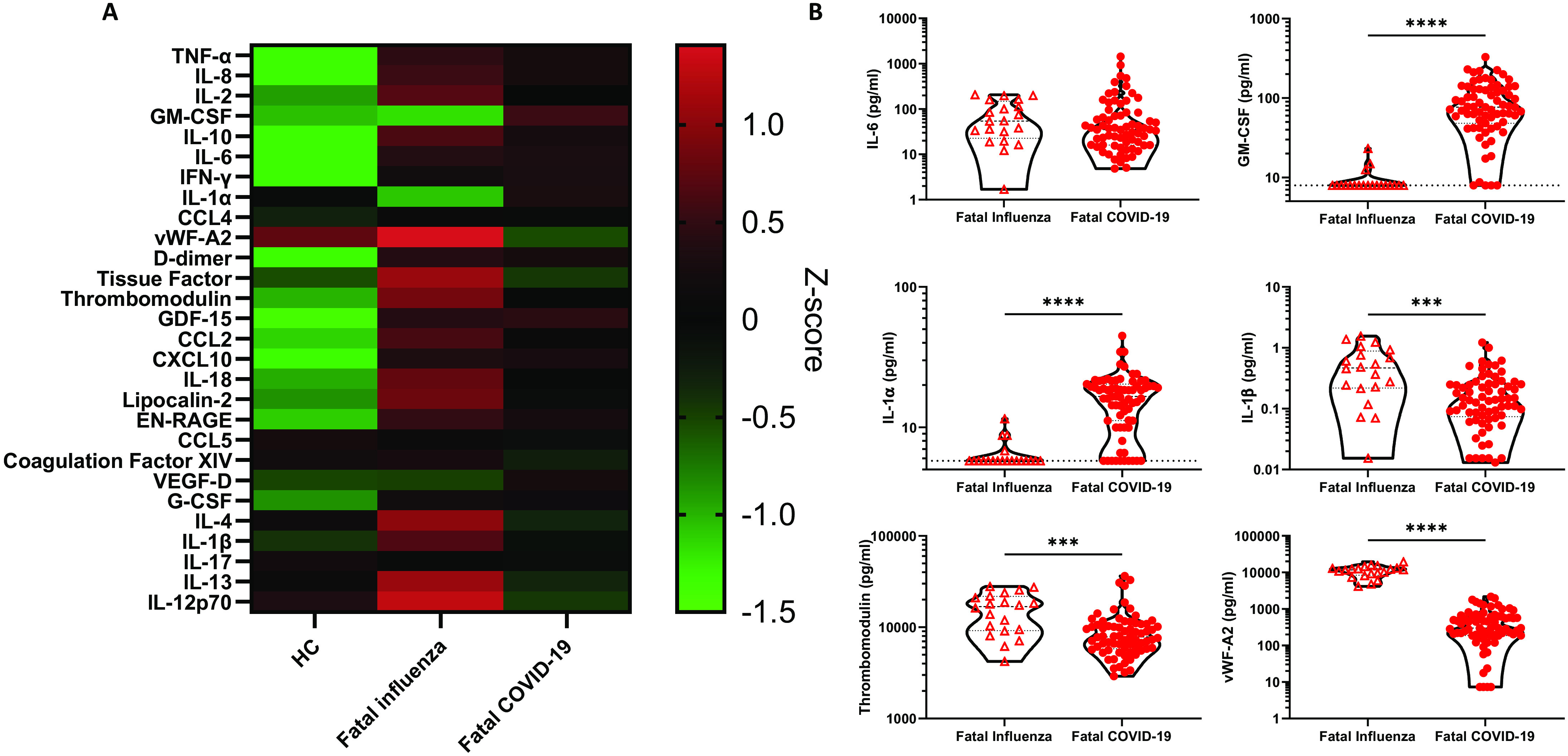
**GM-CSF and IL-1α were elevated in fatal COVID-19 relative to influenza. (A)** Median Z-scores for each mediator between healthy controls (HC, n=15) and patients with fatal influenza (n=20) or fatal COVID-19 (severity 8, n=69). **(B)** Levels of IL-6, GM-CSF, IL-1α, IL-1β, Thrombomodulin, and vWF-A2 in plasma samples from patients with fatal influenza or COVID-19. Data were analyzed for statistical significance using Mann-Whitney tests with between groups. ***P<0.001, ****P<0.0001.

To compare the inflammatory response seen during fatal COVID-19 and influenza, plasma samples from fatal pH1N1 influenza infections (n=20, table S3), collected during the 2009-2011 pandemic, were analyzed on our immunoassay panels. Z-scores were determined between fatal COVID-19, fatal influenza, and healthy controls with the mean Z-score of each group for each mediator ordered according to the hierarchical clustering (seen in Fig. 1). This comparison demonstrated that many of the mediators elevated in fatal COVID-19 were also greatly raised in fatal influenza (Fig. 5A and fig. S7), including IL-1β, Thrombomodulin, and vWF-A2 (Fig. 5B). By contrast IL-6 levels were raised in both groups of patients as shown in other studies (*5*, *41*). However, we found that IL-1α and GM-CSF were significantly elevated in fatal COVID-19 but not in fatal influenza (Fig. 5B), GM-CSF especially distinguishing COVID-19 patients from cases of influenza (fig. S2B).

Given these findings, we sought to determine whether demographic differences between patients with fatal COVID-19 (n=69, table S1) and influenza (n=20, table S3) could account for differences in GM-CSF levels using multiple linear regression. This showed that age and sex were not associated with high GM-CSF levels, which were strongly associated with COVID-19 (*P*<0.0001, table s4). However, chronic cardiac disease (*P*=0.0397) was independently associated with lower GM-CSF levels, likely reflecting the contribution of this risk factor to COVID-19 severity (*26*, *30*) aside from the influence of the inflammatory response. Additionally, diabetes mellitus was independently associated with elevated GM-CSF levels (*P*=0.0220) (table s4), in agreement with previous reports (*42*).

To ensure that these differences in GM-CSF levels were not storage artifacts, we reanalyzed historic data on GM-CSF levels quantified in matched serum samples from these patients, relative to a contemporaneous healthy control cohort (n=36, median age 30.5, 56% male), that we previously made publicly available (*21*). This confirmed that GM-CSF was not significantly elevated in fatal influenza, though a trend was apparent (*P*=0.063, fig. S8). This difference represented a median 1.4-fold increase in GM-CSF relative to healthy controls (medians: HC 1.06 pg/ml; Influenza 1.46 pg/ml), while analysis of plasma samples demonstrated equal medians between healthy controls and fatal influenza (both 7.92 pg/ml) but a 9.7-fold elevation in fatal COVID-19 relative to healthy controls (medians: HC 7.92 pg/ml; COVID-19 76.86 pg/ml). Together, these data support a prominent role for GM-CSF in immunopathology during COVID-19, but not in influenza.

## DISCUSSION

We demonstrated that severe COVID-19 was associated with elevated levels of numerous plasma mediators indicative of coagulation, endothelial activation and a broad inflammatory response including CXCL10, GM-CSF, and IL-6. Amongst these, GM-CSF and IL-1α stood out as being characteristic of COVID-19 and were not found in samples from fatal influenza. Raised levels of many mediators, including GM-CSF, were apparent within the first days of symptoms, potentially indicating a pathologic role for pathways associated with these mediators early in disease.

While markers of fibrinolysis have previously been associated with disease severity (*20*) and thrombosis is common in severe and fatal COVID-19 (*10*, *11*, *24*) the causes of this feature of severe disease are not known. The elevation of angiopoietin-2, thrombomodulin, endothelin-1, and vWF-A2 in fatal COVID-19 cases provides evidence for the involvement of endothelial injury in COVID-19. Endothelial injury following inflammatory damage, including the increasingly recognized pulmonary artery vasculitis (*10*, *24*) in COVID-19, may result in the initiation of a pro-coagulant process involving these cells (*43*). Alternatively, this endothelial injury could be triggered by direct viral infection of vascular cells (though this possibility is uncertain (*43*, *44*), viral replication in non-respiratory tissues is commonly observed at post-mortem (*10*, *13*)); or thrombin mediated activation of IL-1α (*40*). This pro-coagulant role could lead to the deposition of microthrombi, evident in COVID-19 (*10*), activation of the clotting cascade and ultimately elevated D-dimer levels through the degradation of fibrin rich thrombi (*34*).

Neutrophilic inflammation may also contribute to endothelial injury, although neutrophilia is predominantly a feature of the later phases of COVID-19 (*1*) while endothelial injury was evident in the first days of symptoms. However, continued thrombotic events in late-stage fatal COVID-19 may result from neutrophil mediated coagulation as observed in other settings (*45*–*47*) and recently demonstrated in COVID-19 (*48*). Combined, these results indicate a multiplicity of possible pro-coagulant triggers that may contribute to pathology at different stages of disease.

We found that the antiviral immune mediator and leukocyte recruitment factor CXCL10 and the myeloid cell growth factor GM-CSF were strikingly elevated in fatal cases of COVID-19. This is supported by the potential utility of CXCL10 as an early prognostic marker of COVID-19 severity (*49*). An influx of monocytes/macrophages has been described in the lung parenchyma in fatal COVID-19, combined with a mononuclear cell pulmonary arterial vasculitis (*12*), and the presence of pro-inflammatory monocyte-derived macrophages in bronchoalveolar lavage fluid from patients with severe COVID-19 (*10*, *50*). The elevations of CCL2, CXCL10, and GM-CSF in severe disease reported here could contribute to monocyte recruitment and activation leading to this vasculitis, alongside the role of GM-CSF in the recruitment of neutrophils to the pulmonary vasculature (*51*). However, integrative analyses of blood and tissue cellular compartments will be required to determine the sources and functional consequences of the raised levels of these key immunological mediators.

Age is closely associated with COVID-19 severity (*26*, *30*), as observed in our patient cohort. Even allowing for disease severity, we observed elevated levels of inflammatory mediators in older patients relative to younger counterparts. This inflammatory difference may reflect the increasing propensity toward inflammatory responses in older age known as ‘inflammaging’ (*52*). In our study, multivariable analysis indicated that age was not a determinant in the difference in GM-CSF levels between fatal cases of influenza and COVID-19; this suggests that a disease-specific mechanism that exacerbates age-dependent inflammatory responses is likely to occur in COVID-19. Such responses could be determined by many of the epigenetic and metabolic dysfunctions associated with inflammaging (*53*).

Our data indicate that baseline measurement of IL-6 and GM-CSF might allow for stratification of patients into subgroups that might be expected to develop severe disease and benefit from specific anti-cytokine therapies. Based on our data, inhibition of IL-6 may be expected to be equally as effective in influenza as in COVID-19 (*5*, *41*). The benefits of IL-6 inhibition in COVID-19 are not seen in some trials (*54*), but the REMAP-CAP study of critically ill patients with COVID-19 suggests IL-6 receptor inhibition has a place in those with the most severe forms of COVID-19 (*8*). Similarly, the RECOVERY consortium recently demonstrated that biologic IL-6 inhibitors decrease mortality and requirement for invasive ventilation in COVID-19 patients already treated with corticosteroids (*55*).

Our findings support therapeutic targeting of GM-CSF, as previously suggested on theoretical grounds (*56*). Small scale studies of anti-GM-CSF have shown promising results (*57*, *58*) but require formal testing in large clinical trials. One such study, Otilimab in Severe COVID-19 Related Disease (OSCAR; NCT04376684), is on-going. Given the role of GM-CSF in myelopoesis and enhancement of neutrophil survival, alongside the neutrophil activation and dysfunctional myeloid cell populations observed in severe COVID-19 (*59*, *60*), these trials may inform our understanding of the importance of this pathway in COVID-19 immunopathogenesis (*56*).

While early studies demonstrated elevated GM-CSF levels in both ICU and non-ICU treated COVID-19 patients (*1*), we now demonstrate a positive association with disease severity and outcome, in agreement with reports of elevated frequencies of GM-CSF^+^ Th1 cells in patients with COVID-19 requiring ICU treatment (*61*). Additionally, a population of IL-17A and GM-CSF expressing clonally expanded tissue resident memory T cells have been identified in the lungs of patients with COVID-19 (*62*). These studies indicate that pathogenic T cell populations may contribute to the GM-CSF production in patients with severe COVID-19.

One limitation of our study is the lack of a contemporaneous non-COVID-19 ARDS disease control group. This is particularly important for GM-CSF, where analysis of historical plasma samples indicates that elevated levels of GM-CSF appear relatively specific to severe COVID-19. We considered the possibility that prolonged storage may have resulted in the degradation of mediators, though many other cytokines were elevated in these samples relative to COVID-19 (including IL-1β and vWF-A2). However, serum cytokine measurements made at the time of sample collection suggest that elevated GM-CSF is not prominent in cases of severe influenza. A contemporaneous fatal ARDS disease control group would enable direct comparison between infections, but there are currently very few cases of severe influenza due to the non-pharmaceutical measures taken to control COVID-19 (*63*).

The multicenter nature of ISARIC4C adds to the ability to interpret and apply these results to other settings. Further studies are needed to determine the prognostic value of the plasma biomarkers that we identify, alongside markers identified using other methods to enable multivariable analyses of biological data alongside clinical and demographic data. This form of analysis may also enable the phenotyping of patients most likely to respond to individual therapies. The clear early distinction between mediators in patients who progress to severe COVID-19 and those who do not indicates that early therapeutic intervention may be crucial to effective disease modification. We hope that the patterns of responses that we describe will enable rational and novel prognostic and therapeutic approaches to be adopted for controlling COVID-19.

## MATERIALS AND METHODS

### Study Design

The ISARIC WHO Clinical Characterization Protocol for Severe Emerging Infections in the UK (CCP-UK) is an ongoing prospective cohort study of hospitalized patients with COVID-19, which is recruiting in 258 hospitals in England, Scotland, and Wales (National Institute for Health Research Clinical Research Network Central Portfolio Management System ID: 14152) (*64*). The ISARIC4C study aims to comprehensively characterize COVID-19 at the clinical and biological level with the ambition of developing interventions that decrease the morbidity and mortality of COVID-19. Studies so far have defined the clinical risk factors for disease severity and progression (*26*, *30*) and the contribution of host genetics to disease severity (*14*). Future studies seek to define the contribution of viral variants, environmental factors, and the host immune response to disease severity. The protocol, revision history, case report form, patient information leaflets, consent forms and details of the Independent Data and Material Access Committee are available online (*27*). This was a pre-positioned pandemic preparedness study with urgent public health research status (*64*).

### Participants

Hospitalized patients with PCR-proven (n=422, 90%) or high likelihood of SARS-CoV-2 infection (PCR-negative n=12, 3%; no PCR data recorded n=37, 8%) were recruited, including both patients with community- and hospital-acquired COVID-19. This study analyzed EDTA plasma from blood samples obtained on the day of enrollment to the study following a protocol harmonized with international investigators to allow meaningful comparison of results between studies (*25*).

### Study registration and approvals

The ISARIC WHO CCP-UK study was registered at https://www.isrctn.com/ISRCTN66726260 and designated an Urgent Public Health Research Study by the National Institute for Health Research UK. Ethical approval for the ISARIC WHO CCP-UK and this work was given by the South Central - Oxford C Research Ethics Committee in England (Ref 13/SC/0149), the Scotland A Research Ethics Committee (Ref 20/SS/0028), and the WHO Ethics Review Committee (RPC571 and RPC572, 25 April 2013). Healthy controls were recruited prior to December 2019 under approval from the London – Fulham Research Ethics Committee (REC)(reference 14/LO/1023) or from healthy donors following informed consent from a sub-collection of the Imperial College Healthcare NHS Trust National Institute for Health Research Imperial Biomedical Research Centre Tissue Bank. Use of the sub-collection was approved by the Tissue Bank Ethics Committee (Approval R12023). Samples from community managed COVID-19 cases were collected through a subproject of Imperial College London Communicable Disease Research Tissue Bank, under approval from the south central Oxford REC (reference 15/SC/0089). Patients with influenza were recruited between 2009 and 2011, following study approval by the NHS National Research Ethics Service, Outer West London REC (09/H0709/52, 09/MRE00/67) with contemporaneous healthy controls recruited following study approval by the Central London 3 Research Ethics Committee (09/H0716/41), as previously reported (*21*).

### Clinical data collection

A prespecified case report form was used to collect data on patient characteristics, treatments received in hospital and outcomes. A modified Charlson comorbidity index was used to define comorbidities and obesity was clinician-defined. COVID-19 severity was assessed according to the World Health Organization COVID-19 ordinal scale for clinical improvement (*28*). Data were available to report a patient’s maximum illness severity using this scale. To calculate partial HScores (*29*), ferritin, triglyceride and AST measurements from this study were combined with recorded results from case report forms for temperature and routine hemoglobin, white cell counts, and platelet counts.

### Immunoassays

IFN-γ, TNF-α, IL-1β, IL-2, IL-4, IL-6, CXCL8/IL-8, IL-10, IL-12p70 and IL-13 were quantified using MSD (Mesoscale Diagnostics, Rockville, Maryland, USA) V-Plex proinflammatory plates on a SQ120 Quickplex instrument. IL-1α, IL-1ra, IL-6Rα, angiopoietin-1, angiopoietin-2, coagulation factor XIV, endothelin-1, VEGF-D, D-dimer, thrombomodulin, tissue factor, Tie2, von-Willebrand Factor-A2 (vWF-A2), GDF-15, G-CSF, GM-CSF, S100A12/EN-RAGE, IL-17A, IL-18, LCN-2/NGAL, CXCL10/IP-10, CCL2, CCL3, CCL4 and CCL5 were quantified using a Bio Plex 200 instrument (Bio-Rad, Hercules, California, USA) with custom Luminex panel kits from Biotechne (Minneapolis, Minnesota, USA) and MilliporeSigma (Burlington, Massachusetts, USA). IFN-α was quantified in a randomly selected subset of samples as an exploratory analysis using Quanterix (Billerica, Massachusetts, USA) IFN-α assay kits on the SIMOA platform. All values at or below the lower limit of detection (LLOD) were replaced with the geometric mean of the lower limits of detection across plates for each assay. Quantification of GM-CSF in serum samples from influenza cases and controls was performed using MSD.

### Statistical analyses

Statistical analyses used GraphPad Prism v8.3.0 (GraphPad, La Jolla, California, USA) R version 3.6.1 and Python 3.7.3 with Pandas 1.0.3 and Seaborn 0.10.0. The distribution of mediator data was tested by D’Agostino and Pearson normality tests, revealing most data to be non-parametrically distributed. As such these data were analyzed by ANOVA using Kruskal-Wallis tests with Dunn’s test for multiple comparisons of patient groups. Non-parametric two-way analyses were performed using Mann-Whitney U tests. Multiple linear regression analysis was performed in GraphPad Prism using Least squares modelling. Z-scores were calculated using the mean and standard deviation of all values from healthy control, fatal influenza, and fatal COVID-19 groups. Correlation matrix analysis was performed using the R packages ggplot2 and ggcorrplot and Spearman’s test for correlation of non-parametric data, after *P*-value adjustment for multiple testing. Network analysis of inflammatory mediator values using Graphia (version 2.0, Graphia Technologies Ltd (*65*)) was used to visualize mediator-to-mediator correlations based on Spearman correlation coefficients. Edges represent a Spearman correlation coefficient >0.2 (to include all mediators in a single network module) with k-NN edge reduction with k=3. The false discovery rate, or expected proportion of discoveries which are falsely rejected, was controlled using the methods of Benjamini and Hochberg. Heatmaps of log_10_ transformed, scaled and centered plasma mediator data were generated using the ComplexHeatmap package in R with rows and columns split by K-means clustering and dendrograms based on Ward’s minimum variance method (ward.D2) and Spearman’s rank correlations. Linear regression plots for CXCL10 applied an upper limit of detection of 500 pg/ml for consistency across different assay runs. Upper and lower limits of detection (ULOD and LLOD respectively) were applied to cytokine data where values were out of range, particularly for D-dimer (ULOD=11098383 pg/ml), Angiopoietin-2 (ULOD=10101 pg/ml), EN-RAGE/S100A12 (ULOD=312134 pg/ml), Endothelin-1 (LLOD=3.98 pg/ml), IL-2 (LLOD=0.07 pg/ml), IL-4 (LLOD=0.02 pg/ml), IL-12p70 (LLOD=0.06 pg/ml), IL-13 (LLOD=0.73 pg/ml) and IL-17 (LLOD=8.11 pg/ml). The optimal number of K-means clusters was determined by the total within-cluster sum of squares using the ‘elbow’ method in the factorextra package in R. PCA was performed on log_10_ transformed, scaled and centered plasma mediator data using the prcomp function in R. For PCA and heatmap analyses, missing values (totaling 10% of the data set) were imputed by classification and regression trees using the Multivariate Imputation by Chained Equations (MICE) package (*66*). These imputed data were not used for other analyses.

## Supplementary Materials

immunology.sciencemag.org/cgi/content/full/6/57/eabg9873/DC1 Fig. S1 – Routine laboratory parameters, HScores and 4C mortality scores on admission for included hospitalized patients with COVID-19. Fig. S2 – Principal component analysis of all mediator values from the time of recruitment. Fig. S3 – Plasma mediators at the time of enrolment in patients hospitalized with COVID-19. Fig. S4 – Age, but not sex, is associated with differences in plasma cytokine levels within COVID-19 disease severity groups. Fig. S5 – Plasma mediator levels over time. Fig. S6 – Elevated mediator levels are apparent in the first days of symptoms in Severe COVID-19. Fig. S7 – Distinct inflammatory profiles between fatal influenza and COVID-19. Fig. S8 – GM-CSF is not elevated in fatal influenza. Table S1 – Patient characteristics, vital signs and routine laboratory parameters on admission for included hospitalized patients with COVID-19 Table S2 – Clinical characteristics of COVID-19 patient groups identified by clustering of plasma mediator values Table S3 – Clinical characteristics of patients with fatal influenza Table S4 – Multiple linear regression of GM-CSF levels, disease status and demographic variables between patients with fatal COVID-19 and influenza Table S5 – Raw data file (excel spreadsheet) The ISARIC4C investigators
